# Microbial community characterization of shrimp survivors to AHPND challenge test treated with an effective shrimp probiotic (*Vibrio diabolicus*)

**DOI:** 10.1186/s40168-021-01043-8

**Published:** 2021-04-12

**Authors:** Leda Restrepo, Cristóbal Domínguez-Borbor, Leandro Bajaña, Irma Betancourt, Jenny Rodríguez, Bonny Bayot, Alejandro Reyes

**Affiliations:** 1 Department of Biological Sciences, Universidad de los Andes, Bogotá, Colombia; 2 Max Planck Tandem Group in Computational Biology, Universidad de los Andes, Bogotá, Colombia; 3 Escuela Superior Politécnica del Litoral, ESPOL, Centro Nacional de Acuicultura e Investigaciones Marinas, CENAIM, Campus Gustavo Galindo Km 30.5 Vía Perimetral, P.O. Box 09-01-5863, Guayaquil, Ecuador; 4 Escuela Superior Politécnica del Litoral, ESPOL, Facultad de Ciencias de la Vida, FCV, Campus Gustavo Galindo Km 30.5 Vía Perimetral, P.O. Box 09-01-5863, Guayaquil, Ecuador; 5 Escuela Superior Politécnica del Litoral, ESPOL, Facultad de Ingeniería Marítima y Ciencias del Mar, FIMCM, Campus Gustavo Galindo Km 30.5 Vía Perimetral, P.O. Box 09-01-5863, Guayaquil, Ecuador; 6 Center for Genome Sciences and Systems Biology, Department of Pathology and Immunology, Washington University in Saint Louis, Saint Louis, MO USA

**Keywords:** Acute hepatopancreatic necrosis disease (AHPND), *Vibrio diabolicus*, Probiotics, Metagenomics, Comparative genomics, Microbiome, Horizontal gene transfer (HGT)

## Abstract

**Background:**

Acute hepatopancreatic necrosis disease (AHPND) is an important shrimp bacterial disease caused by some *Vibrio* species. The severity of the impact of this disease on aquaculture worldwide has made it necessary to develop alternatives to prophylactic antibiotics use, such as the application of probiotics. To assess the potential to use probiotics in order to limit the detrimental effects of AHNPD, we evaluated the effect of the ILI strain, a *Vibrio* sp. bacterium and efficient shrimp probiotic, using metabarcoding (*16S rRNA* gene) on the gastrointestinal microbiota of shrimp after being challenged with AHPND-causing *V*. *parahaemolyticus*.

**Results:**

We showed how the gastrointestinal microbiome of shrimp varied between healthy and infected organisms. Nevertheless, a challenge of working with AHPND-causing *Vibrio* pathogens and *Vibrio*-related bacteria as probiotics is the potential risk of the probiotic strain becoming pathogenic. Consequently, we evaluated whether ILI strain can acquire the plasmid pV-AHPND via horizontal transfer and further cause the disease in shrimp. Conjugation assays were performed resulting in a high frequency (70%) of colonies harboring the pv-AHPND. However, no shrimp mortality was observed when transconjugant colonies of the ILI strain were used in a challenge test using healthy shrimp. We sequenced the genome of the ILI strain and performed comparative genomics analyses using AHPND and non-AHPND *Vibrio* isolates. Using available phylogenetic and phylogenomics analyses, we reclassified the ILI strain as *Vibrio diabolicus*. In summary, this work represents an effort to study the role that probiotics play in the normal gastrointestinal shrimp microbiome and in AHPND-infected shrimp, showing that the ILI probiotic was able to control pathogenic bacterial populations in the host's gastrointestinal tract and stimulate the shrimp’s survival. The identification of probiotic bacterial species that are effective in the host’s colonization is important to promote animal health and prevent disease.

**Conclusions:**

This study describes probiotic bacteria capable of controlling pathogenic populations of bacteria in the shrimp gastrointestinal tract. Our work provides new insights into the complex dynamics between shrimp and the changes in the microbiota. It also addresses the practical application of probiotics to solve problems with pathogens that cause high mortality-rate in shrimp farming around the world.

Video Abstract

**Supplementary Information:**

The online version contains supplementary material available at 10.1186/s40168-021-01043-8.

## Introduction

Bacterial pathogens cause considerable economic losses and constitute one of the biggest threats for sustainability of aquaculture worldwide [1, 2]. Currently, one of the strongest emerging diseases for shrimp-producing countries is the acute hepatopancreatic necrosis disease (AHPND) [3]. The disease is caused by bacteria of the genus *Vibrio* harboring plasmids (pV-AHPND) that encode genes for the toxins PirA and PirB (*Pir*^*VP*^) [4]. AHPND causes high mortality through a severe peeling of the hepatopancreatic cells, leading to the shrimp’s death [5, 6]. This mortality leads to considerable economic losses in the shrimp farming industry. The extensive and excessive use of antibiotics has increased as a prophylactic treatment to control bacterial diseases in shrimp farms [7]. The systematic use of antibiotics is known to promote antibiotic resistance and remove beneficial microorganisms, which are essential for the healthy development of aquatic animals [7]. Consequently, there is a trend towards imposing more strict regulations on antibiotic use in aquaculture and to reduce the presence of antibiotic residues in aquaculture products [8]. Probiotics have thus emerged as a promising alternative to antibiotics and a way to treat and prevent cultured animal diseases [1].

Probiotics are microorganisms considered to provide beneficial effects to their hosts, either by preventing colonization of pathogenic bacteria through antagonism or promoting animal health through the stimulation of the immune system [1]. Current research in aquaculture focuses on establishing adequate standards on probiotics administration as well as providing an understanding on probiotics’ effects on host-associated microbiota structure and health effects. For example, recent studies in humans demonstrated that the probiotic supplementation restored the normal microbiota composition and function in antibiotic-treated and cesarean-born infants [9]. Another study examined the effect on microbiome recovery after antibiotic therapy on humans, showing an increase in alpha diversity in the microbiomes of the group treated with probiotics [10]. There is thus a need to understand the roles of the microbiota in the health, growth, and survival of cultured aquaculture organisms. Studies in shrimp evaluating gastrointestinal microbial dynamics using probiotics are limited. One of the few studies available is that of Pinoargote et al. [11], where it was shown that probiotics had the ability to keep stable the microbiota in the shrimp’s gastrointestinal tract, which could have positive effects on shrimp survival against AHPND. However, it is still needed to identify beneficial groups of microorganisms among the normal resident microbiota and the mechanisms underlying the interactions with probiotics in the gastrointestinal tract, in order to use them to help improve shrimp’s health.

The choice of probiotics can be controversial in some cases, particularly in aquaculture, where certain probiotic genera are phylogenetically closely related to pathogens [12]. For example, in Ecuadorian shrimp hatcheries, beneficial strains belonging to the species *Vibrio alginolyticus* and AHPND-causing strains of the species *Vibrio parahaemolyticus* belong to the same phylogenetic clade, the *Harveyi* clade, and have been isolated from both, healthy and non-healthy larvae cultures [13]. In particular, a strain previously known as *V*. *alginolyticus* ILI strain was isolated from healthy shrimp larviculture [14]. Since then, it has been demonstrated to be an efficient probiotic agent through in vitro and in situ experiments [12, 14–16]. The *Vibrio* genus is a large and complex taxon that includes free-living bacteria of aquatic environments with the potential to reside in different animal hosts, including the gut and stomach of aquatic animals. The presence of related species with different adaptation strategies within a confined space under strong selection, such as different *Vibrio* species inside the gut of a crustacean, leads to the perfect setup for Horizontal Gene Transfer (HGT) events. This scenario can turn a previously commensal bacterium into a pathogen. We have recently shown the capacity of *Vibrio* to conjugate plasmids, even within distinct clades, and in consequence the transfer of pathogenic characteristics, including the infective capacity of AHPND [17]. pV-AHPND has been identified not only among *V*. *parahaemolyticus* strains, but also in other related species: *Vibrio harveyi*, *Vibrio owensii*, and *Vibrio campbellii* [5, 16, 18–20], within the *Harveyi* clade, and *Vibrio punensis* of the *Orientalis* clade [17].

Although the potential HGT of pathogenicity traits coded in plasmids may be essential to transfer pathogenic properties to genetically related bacteria, AHPND progression does not depend solely on the presence of toxins encoded by the plasmid pV-AHPND. Li et al. [18] studied the repertoire of associated virulence factors in AHPND-causing *V*. *parahaemolyticus* by means of a comparative genomic analysis using the clinical strain RIMD22106 and both, environmental strains not producing AHPND, as well as toxic isolates producing AHPND. The authors described two secretion systems (type III and VI) present in two copies in the pathogenic strains. Specifically, T3SS1 is present in all sequenced strains of *V*. *parahaemolyticus*. It contains 49 genes located on chromosome I [18], including one putative effector protein VstL (homologous to SctL) that is critical for injectosome recruitment and could help with rapid cell death against multiple eukaryotic cell lines [18]. In addition, T3SS2 is composed of 12 genes, two of which (*trh* and *tdh*) share 70% identity with each other and are also present in the human pathogenic strains in *V*. *parahaemolyticus*. TDH is a pore-forming toxin and TRH is a thermolabile toxin and immunologically similar to TDH [19]. All AHPND-causing strains, but none of the non-AHPND strains, have been described to harbor the antibacterial type VI secretion system 1 (T6SS1), which was previously identified and characterized in the clinical isolate RIMD2210633 and is characterized by containing 42 genes [18]. This finding suggests a fitness advantage provided by the T6SS1 acquisition of AHPND-causing *V*. *parahaemolyticus* over competing bacteria, which could facilitate shrimp infection. Thus, the diversity of AHPND-causing pathogens and the different pathogenicity factors they may harbor raises the question of whether other *Vibrio*-related bacteria commonly used as probiotics, such as the ILI strain, could be a safe probiotic or whether or not it could potentially turn into a pathogenic strain.

We aimed to assess the response of the Shrimp’s microbiome to a challenge with AHPND-causing bacteria and test whether the presence of a probiotic, the genetically related ILI strain, could modify such response. Furthermore, we wanted to evaluate if the phylogenetically related ILI strain was capable of acquiring the plasmid pV-AHPND and cause the disease in shrimp. To this end, we performed conjugation assays using a *V*. *parahaemolyticus* strain harboring the pV-AHPND plasmid as a donor and the ILI strain as a potential recipient. Challenge tests were performed on the *Penaeus vannamei* shrimp. Finally, we aimed to better understand the genomic and ecological characteristics of the ILI probiotic and their implications on the interaction with AHPND-causing *V*. *parahaemolyticus*. Thus, we sequenced its genome and performed a comparative genomics analysis with a selected set of AHPND and non-AHPND causing *V*. *parahaemolyticus* isolates, as well as non-AHPND ILI strain-related isolates. Our findings suggest that the ILI strain is a safe probiotic and can be used in AHPND conditions due to the absence of pathogenicity, even after the acquisition of the pV-AHPND plasmids.

## Materials and methods

### Ethics statement

All procedures were approved by the Ethics and Animal Welfare Committee of ESPOL and CENAIM.

### Growth condition of the probiotic strains

We used the ILI probiotic strain provided by the Microbiology Laboratory at the Centro Nacional de Acuicultura e Investigaciones Marinas (CENAIM), previously identified as *V*. *alginolyticus* [13] and characterized as an efficient probiotic [12–16]. This strain was originally isolated from water of a healthy larvae hatchery and has been part of the CENAIM culture collection for over 26 years. The ILI strain was aerobically grown in TSA (Tryptic Soy Agar) with 2% NaCl by seeding and incubated for 24 h at 28 °C. Single colonies were selected and transferred to new TSA plates with 2% NaCl for the conjugation assays [20].

The *Bacillus* sp. strain P64 was originally isolated from wild adult shrimp and has been part of the culture collection at CENAIM since 2004. This strain has been reported to have positive probiotic effects [13–16]. Growth of this strain was performed on marine agar with 2% NaCl as described previously [15].

### Preparation of probiotics inoculum

To generate the inoculum, bacterial probiotic strains were streaked in LB agar with 2% NaCl and incubated for 18 h. One colony for each strain was used to inoculate 10 mL of LB broth. Successive inoculations were made to scale up the volume. Absorbance was adjusted to optical density (OD) at 540 nm, approximately 2 × 10^5^ CFUs (ILI strain) and 2 × 10^8^ CFUs (*Bacillus* strain). Each bacterium was added by spraying daily in the commercial food (~ 35% protein) and immediately administered with the feeding to the shrimp.

### Immersion challenge test of shrimp supplemented with probiotics

Shrimp were supplemented for a 1-month period with the ILI and the *Bacillus* probiotic strains and then challenged with the AHPND-causing *V*. *parahaemolyticus* BA94C2 strain to further evaluate its effects on the microbial community. The BA94C2 strain was isolated from diseased organisms in South America and belongs to the Centro Nacional de Acuicultura e Investigaciones Marinas culture collection since 2015. Four treatments were evaluated. Shrimp that did not receive probiotic and were challenged only with media without bacterium (healthy controls; C0), shrimp that did not receive probiotics but were challenged (C1), shrimp that received the ILI probiotic and were challenged (C2), and shrimp that received the *Bacillus* sp. strain P64 probiotic and were challenged as well (C3). The latter was included to compare its mortality with the ILI probiotic at the end of the challenge test with AHPND. Probiotics were administered for 1 month using the treated feed twice a day (water salinity ~ 34 g l^−1^ and temperature ~ 28 °C). The commercial feed dose used was 3% of the average body weight of the shrimp (adjusted according to the food consumed). Environmental parameters such as salinity, temperature, and dissolved O_2_ and were monitored daily in ponds and tanks after the challenge. Water samples from each treatment were collected before adding probiotics from each tank to evaluate the presence of the probiotic in the tank.

After feeding the shrimp with the corresponding probiotic treatments, survival of the animals to a challenge with *V*. *parahaemolyticus* BA94C2 strain was evaluated. Completely randomized design was applied with four treatments and six replicates per treatment. Experimental units (40 L tanks) were stocked with 40 *P*. *vannamei* shrimp (average weight = 2.5 ± 0.3 g). During the assay, shrimp were monitored for mortality every 2 h. Difference of cumulative mortalities at 50 h post infection among infected treatments were analyzed with the Kruskal-Wallis test using the R software [21]. Shrimp survival was recorded at the end of the experiment. The water exchange (50%) was performed daily before and after the challenge was performed, including during the period of probiotic feeding, but not the 50 h challenge period. Shrimps were exposed to a 12 h photoperiod per day. Pairwise comparisons using Nemenyi test were conducted to evaluate the status (healthy, diseased and with probiotics) to bacterial community variation using R software. Mortalities were expressed as mean ± standard error.

### Re-isolation of bacteria

Shrimp from all treatments were collected at 12 h post infection. Hepatopancreas and stomachs were removed from shrimp and macerated in sterile saline solution. Serial dilutions of 100 μL (10^−1^, 10^−2^ and 10^−3^) containing macerated hepatopancreas and stomachs were inoculated on TCBS agar and incubated at 28 °C for 18 h, and the three most predominant colonies were purified on TSA plates. Afterwards, the DNA of the colonies was extracted and stored for subsequent comparisons with the tested isolates using *Pir*^*VP*^ gene detection (Table S1), as described below. Macerates of each shrimp hepatopancreas and stomachs of healthy controls (C0) were also analyzed for *Pir*^*VP*^ gene detection.

### Metagenomics sample collection

Moribund shrimp exhibited the usual clinical and pathological symptoms of the AHPND, such as lethargy, empty intestine, and pale hepatopancreas. Stomachs and hepatopancreas from each shrimp were aseptically dissected. The tissues were preserved individually in RNA-later solution and stored at − 80 °C until subsequent DNA extraction. After performing the sequencing quality evaluation and trimming (see below), a total of 41 samples of *P*. *vannamei* shrimp were retained—18 from the healthy shrimp treatment (nine hepatopancreas and nine stomachs) and 13 AHPND-infected shrimp not treated with probiotics (eight stomachs and five hepatopancreas)—and five samples from each of the probiotic-treated groups—4 hepatopancreas and 1 pool of 5 stomachs. The stomach tissue of the surviving shrimp was pooled due to the low biomass recovered.

### *16S rRNA* gene sequencing

DNA was extracted from stomachs and hepatopancreas using the PureLink Genomic DNA Mini Kit (Invitrogen; catalog no. K1820-00) according to the manufacturer instructions. DNA was quantified using a Qubit fluorometer HS assay Kit (Life Technologies). The hypervariable V4 region of the *16S rRNA* gene was sequenced using the 515 F/806 R PCR primers [22]. Indexed amplicons were pooled and sequenced on the Illumina MiSeq platform, with 2 × 250 bp paired-end reads. A total of 48 samples were sequenced at Macrogen by using MiSeq 2000 (Illumina, San Diego). Data generated has been deposited in GenBank under BioProject accession number PRJNA580262.

### Data pre-processing and ASV picking

Amplicon sequence variants (ASV) were analyzed with QIIME2 version 2019.1 [23]. Reads were imported into QIIME2 and, subsequently, forward and reverse amplicon sequences were merged with an overlap of 200 bp and demultiplexed according to sample specific indexes, and redundant sequences were removed. Sequences were filtered and denoised into features with DADA2 [24]. In order to obtain a comprehensive description of the within-sample bacterial community, alpha diversity metrics (species richness, Chao1, Shannon diversity and total number of observed OTUs) and rarefaction plots with 3000 sequences per sample were generated. Pairwise comparisons using Wilcoxon rank sum tests were conducted to quantitatively evaluate ASV variations between treatments of healthy, diseased and treated with probiotics, using R software [21]. Likewise, multivariate analyses of beta diversity (Bray–Curtis, Weighted and Unweighted UniFrac) were conducted to assess differences in bacterial community compositions between samples from (a) healthy shrimp (stomach and hepatopancreas), (b) healthy and AHPND-infected shrimp (C0 and C1), and (c) shrimp treated with the two probiotic treatments (C2 and C3). In all cases, we considered taxa accounting for a cumulative 96% of total abundance. In order to visualize these differences, we performed principal coordinate analyses (PCoA) calculating the Bray-Curtis dissimilarity (Beta diversity). Taxonomic assignments of representative sequences were conducted using the Silva database as reference to classify *16S rRNA* gene OTUs clustered at 99% similarity [25]. Differential abundance of taxa was determined based on changes to the Shannon diversity estimates. To determine taxa significantly enriched in healthy shrimp and shrimp treated with probiotics, we applied a linear discriminant (LDA) effect size (LEfSe) analysis method [26].

### Conjugation assays and evaluation of the pathogenicity of the ILI transconjugated bacterial strain

The ILI strain was used as the recipient strain in conjugation experiments. The *V*. *parahaemolyticus* BA94C2 strain harboring the plasmid pV-AHPND [27] was used as donor. To evaluate the capacity of conjugation, experiments were designed following the protocol suggested by Llosa et al. [20] under controlled laboratory conditions simulating natural microenvironments. Control mattings in nutrient TSB broth were performed avoiding contact between strains. Both donor and recipient strains were incubated overnight in LB agar with 2% NaCl at 42 °C and diluted in NaCl to approximately 10^8^ cells per mL by using McFarland’s nephelometer standard. One milliliter of broth containing the donor strain was mixed with 1 mL of broth containing the recipient strain, plus 2 mL of TSB (tryptic soy broth), and incubated for 24 h at 28 °C. After incubation, the conjugation mixtures were diluted 10-fold in 0.9% saline solution to a 10^−7^ dilution. For each sample, 0.1 mL of the dilution was spread on a TSA agar plate with 2% NaCl. The colonies were differentiated by the clear morphological characteristics of the ILI colonies (smaller, with growth following a filamentous shape) and BA94C2 donor (convex, entire and well-defined colonies in appearance, with a size of 7–10 mm in diameter). Colonies were incubated at 28 °C, and then counted and replicated. The presence of the pV-AHPND plasmid and, hence, the detection of transconjugants was performed by PCR amplification of the *PirA* and *PirB* plasmid genes [27] as explained below.

The pathogenicity of the ILI transconjugant bacterial strain was evaluated by an immersion challenge test. Briefly, a single colony of the ILI transconjugant strain was picked and re-suspended in 30 mL of sterile TSB. The culture was incubated for 18 h at 28 °C on a rotary shaker (200 rpm), and the bacterial density was determined on a microplate reader (Varioskan™, Thermo Fisher Scientific). One mL of this solution was transferred to 50 mL of TSB medium and cultured as described below for 10 h. The suspension was plated onto TSA plates after serial dilutions to determine the colony-forming units (CFU) of the isolate. The *V*. *parahaemolyticus* BA94C2 [28] strain was used as positive control under the same conditions. Shrimp were obtained from a pond without the symptomatology of AHPND disease, and the challenge was performed as described by Tran et al. [5], with minor modifications as follows. For the challenge test, transconjugant strains were cultured in TSB for 12 h at 28 °C, until bacterial density reached 2 × 10^9^ CFU mL^−1^. Then, a 15-min immersion was performed on tanks containing 40 healthy shrimp (2.5 ± 0.5 g) per flask and a solution of bacterial suspension with sterile saline water, to achieve a bacterial density of approximately 2 × 10^8^ CFU mL^−1^. Immersed animals were transferred into experimental aquariums filled with 40 L of filtered and UV sterilized seawater and continuously aerated. After transfer, a solution of bacterial suspension was immediately added directly to the experimental tanks containing clean seawater (filtered and UV sterilized), to reach an approximate bacterial density of 2 × 10^6^ CFU mL^−1^. A negative control which consisted of 40 shrimp immersed in sterile TSB, without bacterial strains, was also included. All treatments, including the control, had 10 replicates. During the assay, shrimp were monitored for mortality observations every 2 h. Difference of cumulative mortality at 50 h post infection among infected treatments was analyzed with Wilcoxon’s rank sum test by using the R software [29]. Mortalities were expressed as mean ± standard error.

### Bacterial culture DNA extraction

DNA was extracted with the PureLink Genomic DNA Mini Kit (Invitrogen; catalog no. K1820-00) according to the manufacturer’s instructions with the following modifications: (a) 1 mL of a bacterial culture grown overnight was centrifuged at 4000 rpm for 10 min. The supernatant was removed, and 1 mL of digestion buffer was added. (b) PureLink Genomic Digestion Buffer from the PureLink Genomic DNA Mini Kit and proteinase K (final concentration 2 mg/ml) was added and incubated at 55 °C for 2 h, followed by centrifugation at 5000 rpm for 2 min. (c) After the DNA was cooled at room temperature, it was extracted using the PureLink Genomic DNA Mini Kit (Invitrogen; catalog no. K1821-04) based on binding of the DNA to silica columns, in accordance with the manufacturer’s instructions. (d) The supernatant was discarded, and the samples were air dried for 3 h. DNA extracts were stored at − 20 °C for further analysis. DNA concentrations were determined using a NanoDrop 8000 Spectrophotometer (NanoDrop 2000, Thermo Fisher Scientific Inc.).

### *Pir*^*VP*^ gene detection

The presence of *Pir*^*VP*^ was tested on 152 colonies of donors, recipients, and isolated bacterial strains of transconjugants using a nested PCR method, with primers previously described to obtain amplified products of 1269 and 230 bp (Table S1). Likewise, *PirA* and *PirB* genes were amplified separately with specific primers aimed at generating PCR products of 284 and 392 bp for *PirA* and *PirB*, respectively (Table S1). PCR was carried out in a 10 μl reaction volume containing 10 mM Tris-HCl, pH 8.3, 50 mM KCl, 3 mM MgCl2, 100 mM of each dNTP, 0.4 mM of the specific primer, 2 μl of genomic DNA, and 1 U/μl Taq DNA polymerase (Invitrogen). Amplifications were performed using a Thermo Fisher thermoblock under the following conditions: 4 min initial denaturation at 94 °C, followed by 39 cycles of 5 s denaturation at 94 °C, 45 s annealing at 46 °C, and 1 min elongation at 72 °C, with a final elongation step at 72 °C for 5 min (13). The PCR products (5 μl) were analyzed on a 1.5% agarose gel stained with SYBR Safe DNA Gel Stain (Thermo Fisher), visualized under UV transillumination and photographed using an E-Gel Imager (Thermo Fisher).

### Genome sequencing and de novo assembly

To sequence the genome of the ILI strain, genomic DNA was prepared according to a guide for preparing SMRTbell template for sequencing on the PacBio RS System developed by Pacific Biosciences (catalog number: 100-938-900). The template was sequenced in Macrogen Korea using SMRT Sequencing. SMRTbell^TM^ hairpin loop adapters were ligated to both ends of the double stranded DNA insert to produce a SMRTbell^TM^ sequencing template. Adapter sequences were removed from a CCS (or consensus circular sequencing) read and the read was partitioned to form one or more subreads, which contained the sequence from a single pass of a polymerase on a single strand of an insert within a SMRTbell^TM^. The subreads contained the full set of quality values and kinetic measurements. Reads were trimmed to include only the high-quality regions. Reads containing ambiguous base calls or barcode errors were discarded. De novo assembly was performed using Canu [30]. The complete genome sequence was deposited at DDBJ/EMBL/GenBank under the BioProject accession number PRJNA580299.

### Genome annotation

The genome of the ILI strain was annotated using Prokka and RAST [31, 32]. All predicted ORFs were translated and searched (BLAST) against (1) non-redundant protein databases from NCBI (E-value threshold < 10^−6^ and minimal alignment length ≥ 80%), (2) Swiss-Prot, (3) Clusters of Orthologous Groups (COG), (4) Kyoto Encyclopedia of Genes and Genomes (KEGG), and (5) Gene Ontology (GO) [33–35].

### Multilocus sequence typing phylogeny

A multilocus sequence typing (MLST) analysis was performed with the phylogenetic marker genes: *ftsZ*, *gapA*, *gyrB*, *mreB*, *topA*, and *16S rRNA*, from a dataset of 78 related *Vibrio* species obtained from the NCBI Reference Sequence Database (RefSeq). GenBank accession numbers are listed in Table S2. We used the TCS program [36] to calculate a phylogenetic network estimation among *Vibrio* species used for the MLST analysis and to choose the most related strain to the ILI strain. Sequences were aligned with MEGA 6.0 [37] using the MUSCLE algorithm. Phylogenetic trees were constructed using maximum likelihood (ML). We use JModeltest 2.0 [38] to test the evolution models based on the hierarchical likelihood ratio test, determining that the TIM+G model best fit the data.

### Comparative genome analysis and genomic island identification

MAUVE software [39] was used to perform whole genome alignments and comparisons. The genome of the ILI strain was aligned to the two most cured, well-annotated, and complete genomes of the related species (*V*. *alginolyticus* strain NBRC 15630 = ATCC 17749 and *V*. *antiquarius* strain EX25, accession numbers: GCA 000354175.2 and GCA_001909305.1, respectively) to evaluate gene conservation. Genes previously predicted using Pfam for the identification of genomic islands (GIs) and several common characteristics of the genome, such as abnormal sequence composition and the presence of mobile genetic elements were used [40].

### Average nucleotide identity, correlation indexes and DDH estimates

Full genome comparison statistics were determined with the Genome-to-Genome Distance Calculator (GGDC 2.1, http://ggdc.dsmz.de/distcalc2.php). The average nucleotide identity (ANI) was measured between pairs of genomes, based on the BLAST algorithm (ANIb). In addition, we calculated the tetranucleotide frequencies (TETRA) for the different genomes. The ANIb and TETRA values of the *Vibrio* genome comparisons were calculated for all possible bacterial pairs, including all species identified in the GenBank database as *V*. *antiquarius* (three species), 16 species of *V*. *alginolyticus*, and the ILI strain. All comparisons were represented as heatmaps using the R statistical software [21].

## Results

### Probiotics, in particular ILI strain prevents dysbiosis of the microbiota caused by AHPND

We evaluated the changes of the microbial community structure caused by AHPND on shrimp stomachs and hepatopancreas, with and without the presence of probiotics. Shrimp were either not challenged (healthy controls; C0) or challenged with AHPND causing *V*. *parahaemolyticus* BA94C2 strain (AHPND-infected controls; C1) and compared with the same setup but with the daily supplementation of a probiotic (via feeding in the commercial food without other probiotic medication) for a 1-month period prior to the challenge. We used as probiotic the ILI strain (C2) and another probiotic (*Bacillus* sp. strain P64) for comparison (C3). The results from the challenge test showed the highest survival values for treatments C0 and C2 (*P* < 0.001, for both treatments in comparison with the C1 and C3 treatments, Nemenyi test), followed by C3, and finally shrimp that showed the highest mortality were those of the AHPND-infected shrimp treatment (C1) (Fig. S1).

Characterization of the microbiota of stomachs and hepatopancreas through V4 *16S rRNA* gene sequencing was performed using an Illumina MiSeq v3 kit (2 × 250 PE). A total of 58,074,501 high-quality reads were obtained from 42 samples (3000–49,057 reads per sample). Rarefaction curves showed sampling saturation was achieved well before 3000 reads (Fig. S2). In order to remove the minimum number of samples, a rarefaction of the data to 3000 sequences per sample was performed, resulting in a total of 737 ASVs across samples. The final dataset contained information for nine hepatopancreas and nine stomachs of healthy shrimp, five hepatopancreas and eight stomachs of AHPND-infected shrimp, and four hepatopancreas and one pool of five stomachs for each one of the probiotic treatments.

**Fig. 1:**
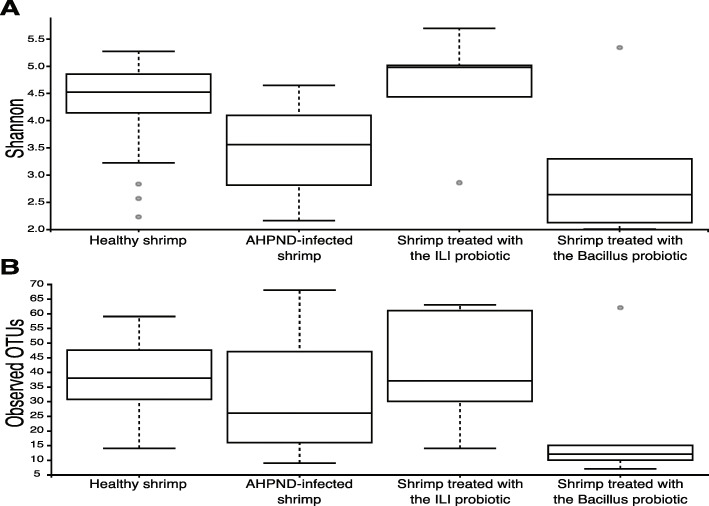
Microbial alpha diversity is reduced by AHPND. Boxplots of two different alpha diversity indexes are shown for all samples grouped by health status **a** Shannon index and **b** number of observed OTUs (ASVs). More variation in the composition of gastrointestinal microbiota for healthy shrimp samples is observed. An increase was observed in the indexes for shrimp treated with the ILI probiotic. There is a stronger effect on diversity (Shannon) than on richness (Observed OTUs) by the treatments

The diversity of the microbiota in healthy shrimp (C0) was significantly higher than on AHPND-infected shrimp (C1), as observed using different alpha diversity metrics (Fig. 1). In particular, Shannon diversity showed a high diversity in shrimp treated with the ILI probiotic (C2), equivalent to that of healthy shrimp (C0) (healthy 4.24 ± 0.89 vs. infected 3.42 ± 0.90; *P* = 0.861, Nemenyi test) in accordance to the mortality rates displayed. Equivalent results were obtained with Chao1 (healthy 934 ± 8.21 vs. infected 822 ± 3.41; *P* = 0.786, Nemenyi test). Furthermore, although the *Bacillus* probiotic (C3) had lower diversity than infected controls (C1), no statistical significance was observed (2.45 ± 0.41, *P* = 0.149, Nemenyi test).

**Fig. 2:**
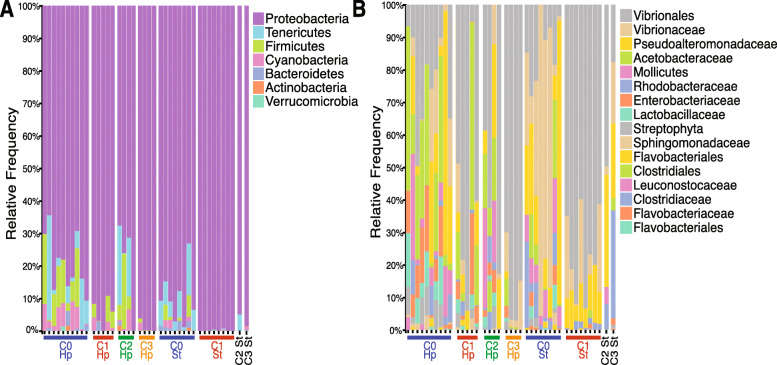
The gastrointestinal microbiome in shrimp is dominated by few Phyla and bacterial families. Relative abundance of **a** phylum and **b** bacterial family for healthy controls (C0), AHPND-infected controls (C1), and shrimp that received ILI probiotic (C2) and *Bacillus* probiotic (C3). Divisions among columns and color bars at the bottom of the figure are used to focus on the differences between organs, hepatopancreas (hp), and stomach (st). The top 7 most abundant phyla are shown, and the top 16 family level classifications are shown. Notice the changes between healthy and infected shrimp and the similarity to the healthy status shown in shrimp treated with the ILI probiotic (C2)

Upon analysis of the microbial community structure, Proteobacteria, Firmicutes, and Tenericutes were the most abundant phyla, i.e., their accumulated abundance is higher than 90% across treatments, both in hepatopancreas (hp) and stomach samples (st) (Table 1). A reduction in the number of phyla was observed in hepatopancreas and stomachs of AHPND-infected shrimp (hp = 12 phyla and st = 4 phyla) compared with healthy animals (hp = 14 phyla and st = 8 phyla), except for shrimp treated with the ILI probiotic, supporting the previous observation regarding alpha diversity. Even though Proteobacteria was the most abundant phyla overall, the rank abundance of the next most abundant phyla varied among different treatments and organs. For example, hepatopancreas from shrimp treated with ILI resemble most closely resemble the rank abundance of healthy shrimp (Table 1, Fig. 2a).

**Table 1:** Values (percent) of relative abundance of the four most abundant phyla for healthy shrimp, AHPND-infected shrimp, and shrimp treated with ILI or the *Bacillus* probiotic

Phyla level								
	Healthy		AHPND-infected		Treated with the ILI probiotic		Treated with the ***Bacillus*** probiotic	
	Hp	St	Hp	St	Hp	St	Hp	St
Proteobacteria	77.95	89.81	94.45	99.84	79.33	94.93	99.08	98.55
Firmicutes	9.38	1.46	3.33	0	7.56	0	0.33	0
Tenericutes	6.89	6.65	0	0.02	10.39	4.92	0	0
Cyanobacteria	4.74	0.73	1.59	0.06	2.03	0	0.65	1.12

At the family level, the most abundant taxa across all samples are shown in Table 2. The most abundant family could be assigned taxonomically at the order level (Vibrionales). Similar to the observation at the phylum level, challenged shrimp showed a reduction in diversity by expansion of the Vibrionales. This was observed in both tissues and treatments, except for ILI treated animals (Fig. 2b). Interestingly, ASVs assigned at family level to the Vibrionales or Vibrionaceae were expanded in particular in the stomach of infected individuals, thus showing that more than one Vibrionales species were increasing their abundance in affected shrimp.

**Table 2:** Values (percent) of relative abundance of most abundant families for Healthy shrimp, AHPND-infected shrimp, and shrimp treated with the ILI and Bacillus probiotic

Family level								
	Healthy		AHPND-infected		Treated with the ILI probiotic		Treated with the ***Bacillus*** probiotic	
	Hp	St	Hp	St	Hp	St	Hp	St
Vibrionales (Order)	18.06	14.95	55.33	75.70	42.15	48.64	87.26	16.65
Pseudoalteromonadaceae	15.94	21.70	5.75	10.31	13.53	32.94	0.58	21.63
Acetobacteraceae	24.69	5.37	17.38	0.36	11.72	0	2.44	3.59
Rhodobacteraceae	3.13	8.62	1.77	1.73	2.51	8.07	0.06	35.81
Enterobacteriaceae	9.38	1.29	7.26	0.16	3.13	0	0.89	1.83
Mollicutes (Class)	6.89	6.65	0	0	10.39	4.92	0	0

**Fig. 3:**
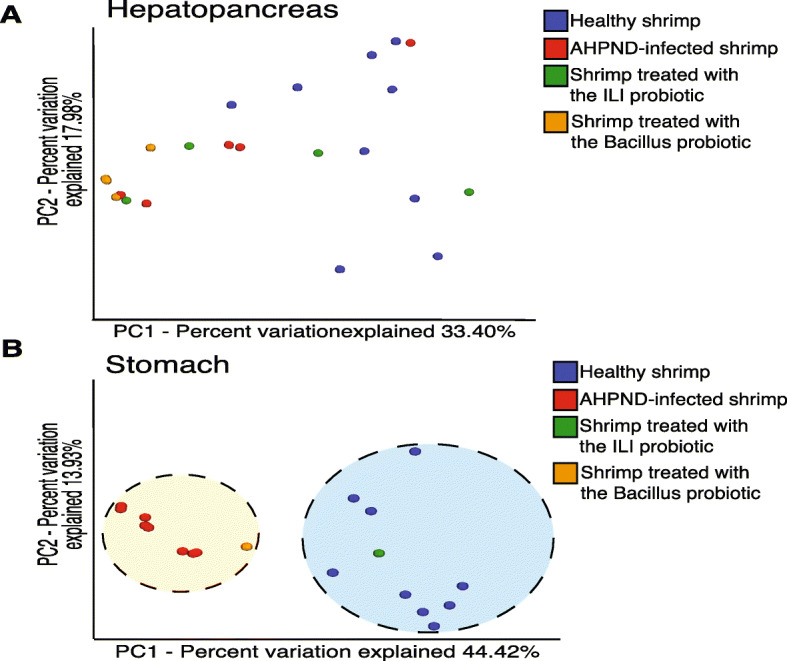
Beta diversity analysis of microbiota from hepatopancreas and stomach samples. **a** A PCoA graphical representation based on Bray–Curtis similarity does not show a clear grouping pattern between different treatments for hepatopancreas samples in the two main axes explaining over 50% of the variation. **b** PCoA plot showing a clear grouping pattern that separates stomachs of infected from healthy samples. Variations between groups highlight the clustering of the ILI probiotic with the healthy shrimp, while the Bacillus probiotic seems closer to the infected individuals. Notice that all samples were ran in the same PCoA analysis but are separated by organ for visualization purposes. Each colored circle corresponds to a type of sample; healthy individuals (blue), infected individuals (red), with ILI probiotic (green) and *Bacillus* probiotic (orange). Probiotic treatments in panel B represent a pool of 5 stomachs processed together

To compare the community structure among treatments, a beta diversity analysis, using the Bray–Curtis dissimilarity metric, was performed. Comparisons between healthy (C0) and AHPND-infected shrimp (C1) showed that there was no clear clustering between treatments in hepatopancreas samples (Fig. 3a). Conversely, stomach samples clustered depending on the health status of shrimp (Fig. 3b), indicating that AHPND infection clearly affects the configuration of the gastrointestinal microbiota. Even though hepatopancreas samples treated with the probiotic did not form a clear cluster, these (C1) showed a reduced distance to the healthy shrimp (C0) compared to the infected animals. In pooled stomach samples, those treated with probiotics had intermediate positions between healthy and infected samples, but ILI-treated samples (C2) grouped closer together with controls (C0), while samples treated with *Bacillus* probiotics clustered together with the infected ones.

**Fig. 4:**
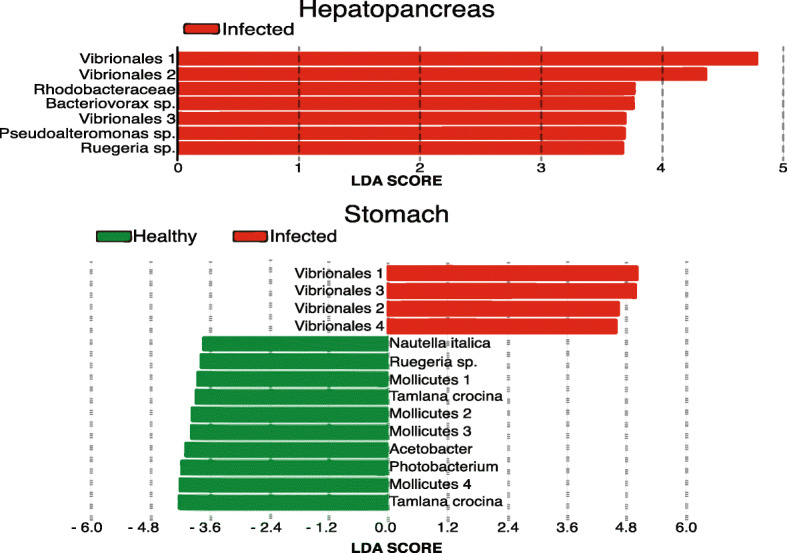
LEfSe results of enriched ASVs of healthy and infected organisms. Enrichment was tested separately for ASVs in hepatopancreas (**a**) and stomachs (**b**). For the hepatopancreas, seven ASVs were the main contributors for the diversity differences observed and were only found enriched on infected individuals. For stomachs, fourteen ASVs showed differential abundance in AHPND-infected compared with healthy shrimps. The graph shows the log10 LDA score for each classification

A linear discriminant analysis effect size (LEfSe) was conducted to determine the significant differences in the abundance of ASVs between healthy and AHPND-infected shrimp. This analysis was calculated for each ASV identified in healthy and infected samples and was used to determine potential biomarkers in each microbiome of the probiotics tested. In hepatopancreas, changes in the relative abundance of seven ASVs were the main contributors to the observed diversity differences, through enrichment on infected individuals (Fig. 4a). It is noteworthy that all seven ASVs were assigned to the Phylum Proteobacteria, and three of these were assigned to the order Vibrionales, but no further taxonomical assignment was possible.

In stomach samples, fourteen ASVs showed differential abundance among treatments. Four ASVs were significantly over abundant in AHPND-infected shrimp (Vibrionales 1, Vibrionales 3, Vibrionales 2, and Vibrionales 4), whereas the remaining ten ASVs were significantly more abundant in healthy shrimp (Fig. 4b). Analogous to what was found in hepatopancreas, only members of the Proteobacteria, and in this particular case of the Vibrionales, were among the disease-related taxa. Taxa enriched in control samples included members of the Tenericutes (4), Proteobacteria (4), and Bacteroidetes (2), reflecting a broader taxonomical diversity in healthy microbiota. Within the 4 Proteobacteria taxa increased in control samples, only one member of the Vibrionaceae family (*Photobacterium damselae*) was found (Table 3), but its potential role in the shrimp’s health is not clear.

**Table 3:** ASVs taxonomical assignment for those identified as differentially abundant in AHPND-infected compared with healthy shrimp

ASV ID	Phylum	Class	Order	Family	Genus	Species	Organ
ASV1	Proteobacteria	Gammaproteobacteria	Vibrionales	–	–	–	Hepatopancreas
ASV2	Proteobacteria	Gammaproteobacteria	Vibrionales	–	–	–	Hepatopancreas
ASV3	Proteobacteria	Alphaproteobacteria	Rhodobacterales	Rhodobacteraceae	–	–	Hepatopancreas
ASV4	Proteobacteria	Deltaproteobacteria	Bdellovibrionales	Bacteriovoracaceae	*Bacteriovorax*	*Bacteriovorax sp*.	Hepatopancreas
ASV5	Proteobacteria	Gammaproteobacteria	Vibrionales	–	–	–	Hepatopancreas
ASV6	Proteobacteria	Gammaproteobacteria	Vibrionales	Pseudoalteromonadaceae	*Pseudoalteromonas*	*Pseudoalteromonas sp*.	Hepatopancreas
ASV7	Proteobacteria	Alphaproteobacteria	Rhodobacterales	Rhodobacteraceae	*Ruegeria*	*Ruegeria sp*.	Hepatopancreas
ASV1	Proteobacteria	Gammaproteobacteria	Vibrionales	–	–	–	Stomach
ASV8	Proteobacteria	Gammaproteobacteria	Vibrionales	–	–	–	Stomach
ASV2	Proteobacteria	Gammaproteobacteria	Vibrionales	–	–	–	Stomach
ASV9	Proteobacteria	Gammaproteobacteria	Vibrionales	–	–	–	Stomach
ASV10	Proteobacteria	Alphaproteobacteria	Rhodobacterales	Rhodobacteraceae	*Nautella*	*Nautella italica*	Stomach
ASV11	Proteobacteria	Alphaproteobacteria	Rhodobacterales	Rhodobacteraceae	*Ruegeria*		Stomach
ASV12	Tenericutes	Mollicutes	–	–	–	–	Stomach
ASV13	Bacteroidetes	Flavobacteriia	Flavobacteriales	Flavobacteriaceae	*Tamlana*	*Tamlana crocina*	Stomach
ASV14	Tenericutes	Mollicutes	–	–	–	–	Stomach
ASV15	Tenericutes	Mollicutes	–	–	–	–	Stomach
ASV16	Proteobacteria	Alphaproteobacteria	Rhodospirillales	Acetobacteraceae	*Acetobacter*	*Acetobacter sp*.	Stomach
ASV17	Proteobacteria	Gammaproteobacteria	Vibrionales	Vibrionaceae	*Photobacterium*	*Photobacterium damselae*	Stomach
ASV18	Tenericutes	Mollicutes	–	–	–	–	Stomach
ASV19	Bacteroidetes	Flavobacteriia	Flavobacteriales	Flavobacteriaceae	*Tamlana*	*Tamlana crocina*	Stomach

Given that taxonomic assignments of ASVs among Vibrionales reached the family rank at best, we evaluated nucleotide dissimilarity among these ASVs in order to identify species- or clade-specific SNPs that will allow a further taxonomic assignment. Specifically, we aimed to determine whether some of these ASVs corresponded to the genus *Vibrio*, which are of great importance in the cultured shrimp microbiota. We identified SNPs that allowed the clustering of related variants. However, the SNPs themselves did not provide enough resolution to resolve at the species level, but the clustering allowed to differentiate between known *Vibrio* clades. The three ASVs of Vibrionales were found within the cluster containing the species from the *Orientalis*, *Coralliilyticus*, and *Harveyi* clades (Fig. S3). A SNP in position 119 of the alignment showed differences between species of the three clades. Species that shared this SNP with the ASV for the *Orientalis* clade were *Vibrio sinaloensis*, *Vibrio hepatarius*, *V*. *orientalis*, and *Vibrio brasiliensis*; for *Coralliilyticus* clade, *Vibrio neptunius*; and finally, for the *Harveyi* clade, *V*. *parahaemolyticus* and *V*. *campbellii*.

Finally, we compared the nineteen ASVs that were differentially abundant in hepatopancreas and stomach samples of infected and healthy shrimp, as potential biomarkers in each of the microbiomes from the probiotic treatments. The most abundant ASVs characterized in hepatopancreas of AHPND-infected shrimp (Vibrionales 1 and Vibrionales 2) were found in different proportions among hepatopancreas microbiomes (Fig. S4A). The Rhodobacteraceae (ASV 3), *Bacteriovorax* sp. (ASV 4), and *Ruegeria* sp. (ASV 7) ASVs were found on AHPND-infected shrimp only, while Vibrionales 3 (ASV 5) was found both in AHPND-infected shrimp and shrimp treated with the *Bacillus* probiotic. When comparing ASVs as markers in stomach samples, we found that the four ASVs whose proportions were over-represented in infected organisms (Vibrionales 1, 2, 8, and 9) were stable across probiotic-treated animals, present at a comparable abundance as that in healthy animals. We observed ten ASVs in healthy shrimp: *Nautella italica*, *Ruegeria*, and Mollicutes 1 (ASVs from 10 to 12) were found in a higher proportion in healthy animals, while the Mollicutes 2, 3, and 4, *Acetobacter* and *Photobacterium damselae* (ASVs from 14 to 18) showed a significant increase in the ILI treatment as compared to healthy animals (Fig. S4B). The role of Photobacterium is unclear; it is known to inhabit many ecological niches and can perform a variety of functions related to host health [41], but it has also been associated with different diseases during shrimp farming [42]. An interesting idea may be to evaluate the role that this bacterium plays during AHPND infection. The finding of ten ASVs observed in healthy shrimp could be associated to the higher survival rate observed in shrimp treated with ILI. *Tamlana crocina* (ASV13) was found in higher proportions in probiotic treatments compared to controls; thus, it may be playing a role in shrimp’s recovery.

### Bacterial bioassay shows that ILI probiotic strains do not cause AHPND in *Penaeus vannamei* shrimp

**Fig. 5:**
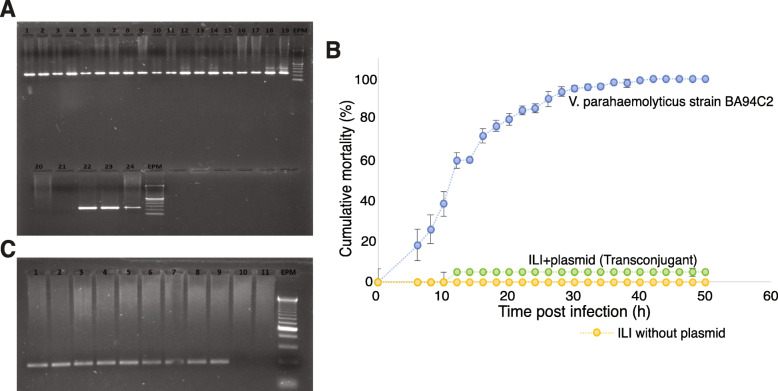
Identification of *Pir*^*VP*^ genes in isolated colonies from ILI strain. **a** PCR characterization of ILI’s individual colonies (1-23) and positive control (lane 24; *V*. *parahaemolyticus* BA94C2). PCR targets the *Pir*^*VP*^ genes coded in the plasmid from BA94C2. Notice that all strains were positive for the plasmid except two (lanes 20 and 21). EPM: DL2000 DNA marker. **b** Shrimp cumulative mortality after being challenged with transconjugant colonies of the ILI strain and *V*. *parahaemolyticus* BA94C2 strains. Negative control was TSB 2% NaCl. Bars indicate standard deviations (*n* = 5 replicates, see methods for the number of shrimps per assay). **c** ILI’s individual colonies recovered from hepatopancreas and stomachs of surviving shrimp challenged with transconjugant colonies of the ILI strain. Lane 1: *V*. *parahaemolyticus* BA94C2 containing the *Pir*^*VP*^ genes; lanes 2–8: ILI’s individual colonies; lane 9: *V*. *parahaemolyticus* BA94C2 containing the *Pir*^*VP*^ genes; lane 10: ILI colony not containing the *Pir*^*VP*^ genes; and lane 11: PCR negative control; EPM: DL2000 DNA marker

Given the positive protective results obtained with the ILI probiotic strain and its close phylogenetic relationship with pathogenic strains, we decided to perform further genomic and experimental analyses on this strain. The *V*. *alginolyticus* ILI probiotic strain is a close relative of the pathogenic *V*. *parahaemolyticus* but does not contain the plasmid pV-AHPND with the *Pir*^*VP*^ genes coding for the AHPND-causing toxins PirA and PirB. We performed conjugation assays to evaluate the capacity of the ILI strain (recipient) to acquire the pV-AHPND plasmid from *V*. *parahaemolyticus* BA94C2 strain (donor). We found a high conjugation frequency of colonies harboring the plasmid carrying the *Pir*^*VP*^ genes, with 70% of the evaluated ILI strain colonies containing the plasmid (Fig. 5a).

Transconjugant ILI colonies were isolated and used as the infecting agent for a challenge test with healthy *P*. *vannamei* shrimp, in order to evaluate their ability to cause AHPND in shrimp. Low mortality was observed for the transconjugant ILI treatment as well as negative controls (Fig. 5b). Shrimp mortality in the positive control (challenged with the *V*. *parahaemolyticus* BA94C2 strain) started after 6 h post-infection, reaching 96.2% ± 0.5% at 50 h post-infection, resulting in a significantly different (*P =* 0.005, Kruskal-Wallis test) mortality compared to the transconjugated ILI strain treatment (Fig. 5b). Shrimp challenged with the transconjugant ILI strain were used for isolating predominant *Vibrio* strains. The recovered ILI strains were still positive for both PirA and PirB toxin genes. All *Vibrio* strains recovered from the macerates of the negative controls remained negative for the toxins (Fig. 5c).

### Genomic features of *ILI* probiotic strain and phylogenetic re-classification as *Vibrio diabolicus*

Given its proven non-pathogenic capacity of the ILI probiotic strain in our essays, even after acquiring the pV-AHPND, we aimed to understand the presence or lack of other potential virulence factors that could partly explain our findings. The genome of the ILI strain was sequenced using long reads (PacBio RS) and assembled into three scaffolds corresponding to chromosomes I (3.26 Mbp) and II (1.83 Mbp), and one large plasmid (340.52 Kbp, G+C content of 40.27%), using a total of 82,331 PacBio reads. The average coverage achieved was 46.3X, with G+C content of 44.6%. We predicted 4,956 coding genes (Table S3) within a total genome size of 5.4 Mbp (Table 4). Remarkably, the large plasmid not only had an unusually low G+C content, but it did not show any significant similarity with plasmids previously described from *Vibrio* genomes available to date in public databases. The most closely related plasmid identified was described in *V*. *campbellii* strain 20130629003S01 with 34% similarity, a size of 204,531 bp and non-annotated function. Among the 496 plasmid proteins predicted by HMMER [43], only 96 (19%) had significant matches in the NCBI non-redundant database (Table S2), leaving a total of 81% of proteins with unknown function.

**Table 4:** Genomic characteristics of the genome strain ILI

Attributes	Values
Assembly size (bp)	5,436,990
Total number of contigs	3
GC content %	44.6
Number of CDS	4,956
Number of rRNAs	18
Number of tRNAs	58

**Fig. 6:**
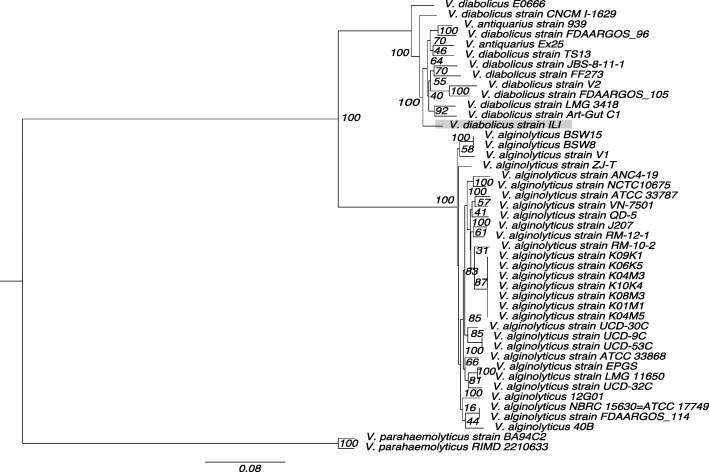
ILI strain reclassification as *V*. *diabolicus*. Phylogenetic analysis of the concatenated alignment based on *ftsZ*, *gapA*, *mreB*, *recA*, *rpoB*, *topA*, and *16S rRNA* genes. A maximum likelihood phylogeny on a concatenated multiple sequence alignment using the TIM+G evolutionary model is shown. Numbers above the branches represent the posterior probabilities and percent bootstrap values (one thousand bootstrap replicates). The tree includes 10 *Vibrio diabolicus* strains, 20 *Vibrio alginolyticus* strains, 2 *Vibrio antiquarius* strains, and 2 *Vibrio parahaemolyticus* strains as the outgroup. Scale bar represents number of substitutions per site. Highlighted in grey is the position of the ILI strain

The ILI probiotic strain was originally classified as *V*. *alginolyticus* [10] based on MLST analysis. We used the full genomic sequence and through comparisons against publicly available genomes (Table S4) showed that the closest significant matches are with *Vibrio antiquarius* EX25 (98% identity) contrasting with a 92% average identity against *V*. *alginolyticus*. To clarify the exact position of the ILI strain within the *Harveyi* clade, we used all available genomes of *V*. *alginolyticus* (31 genomes), *Vibrio diabolicus* (ten genomes) and *V*. *antiquarius* (two genomes) species, and two additional genomes of *V*. *parahaemolyticus* as an outgroup (Table S5). A concatenated alignment of seven genes (*ftsZ*, *gapA*, *mreB*, *recA*, *rpoB*, *topA*, and *16S rRNA*) commonly used in MLST analysis was used to construct a maximum likelihood phylogenetic tree clearly separating *V*. *alginolyticus* from the *V*. *diabolicus* and *V*. *antiquarius* species in the *Harveyi* clade (Fig. 6). The phylogenetic tree revealed a high similarity level of the ILI strain with *V*. *antiquarius* and *V*. *diabolicus*, and four strains (E0666, FF273, TS13, and V2) previously identified as *V*. *alginolyticus* but recently reclassified as *V*. *diabolicus* by Turner et al. [44]. In that same study, it was suggested that *V*. *diabolicus* and *V*. *antiquarius* were the same species, and *V*. *diabolicus* should be used as it was described before *V*. *antiquarius*. Thus, our results clearly separate the ILI strain from the *V*. *alginolyticus* subclade and cluster this strain with others identified as the species *V*. *diabolicus*.

**Fig. 7:**
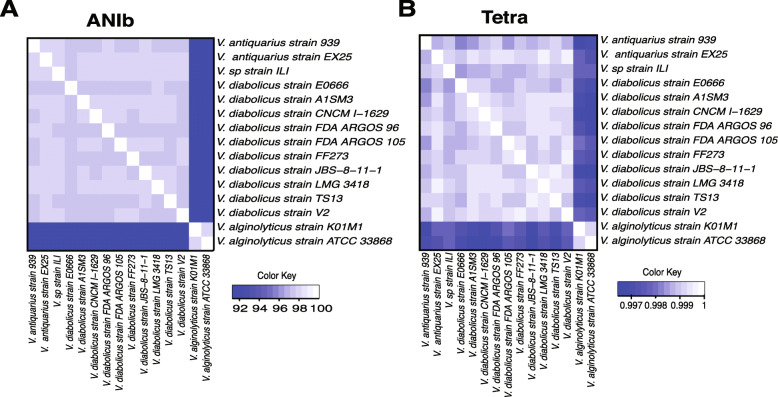
Heatmaps representing phylogenomics metrics. Evaluation of species circumscription among *Vibrio antiquarius*, *Vibrio alginolyticus*, and *Vibrio parahaemolyticus* strains. The extent of nucleotide identity was calculated using ANIb (**a**) and Tetra (**b**) as illustrated. Blue box shows the ILI strain sample. The species and strain number are shown

To further confirm this new classification, we took advantage of the availability of full genome sequences to perform genome-wide comparison against a selected set of representative isolates (Table S6), including AHPND-causing and non-causing strains. The in silico-DDH (DNA-DNA hybridization) analysis showed that the ILI strain displayed a value higher than the traditional species delimiting cutoff (70%) with *V*. *antiquarius* EX25 (83.80%), and below the threshold with *V*. *alginolyticus* species (≤ 46.30% Table S6). In addition, the percentage of differences of the G+C content between ILI strain and *V*. *antiquarius* was < 1%, corresponding to values obtained traditionally among members of the same species [45]. Phylogenomic metrics (ANI and Tetra) [43] were performed and the values between ILI and EX25 strain (reclassified as *V*. *diabolicus*, but still found in the nucleotide databases as *V*. *antiquarius*) was 98.03% for ANIb (Fig. 7a and Table S5). Accordingly, Tetra values indicated that the ILI strain is more related to EX25 strain based on the correlation coefficient of 99.8% (Fig. 7b). Based on the aforementioned results and the traditional thresholds of 95% and 0. 997 for ANI and Tetra to delimit the same species, here we reclassify the ILI strain as *Vibrio diabolicus*.

### Identification of specific virulence features of the *V*. *diabolicus* ILI strain genome

The ILI genome contained 104 homologs of virulence genes found in other *Vibrio* species. Most of the homologs were classified in the subsystems of resistance to antibiotics and toxic compounds [46], which is interesting given the lack of pathogenicity shown in our experimental setups (Table S7). Furthermore, ILI’s genome encodes a number of genes that include cytochrome peroxidase and cytochromes for reduction of oxygen to hydrogen peroxide and superoxide for tolerating high oxygen concentrations.

The presence of known secretion systems has been used to distinguish among *V*. *parahaemolyticus* pathogenic and non-pathogenic strains. In particular, *V*. *parahaemolyticus* contains two copies of type III secretion systems (T3SS1 and T3SS2), and two copies of type VI secretion systems (T6SS1 and T6SS2), where the numbering also signals their locations in chromosomes I and II, respectively. T3SS1 on ILI is also present in chromosome I and contains 43/49 genes (Table S7). In general, most of the genes show 98% average percent identity with the orthologs in *V*. *alginolyticus* and *V*. *diabolicus*, while only 84% with *V*. *parahaemolyticus* (Figure S4). In comparison, when evaluating 8 genes upstream and 9 genes downstream from T3SS1 in *V*. *parahaemolyticus* RIMD 2210633, a total of 15 out of 17 genes were present in ILI, with an average percent identity of 91.71%.

T3SS2 is known to be present in clinical isolates but absent from environmental non-AHPND and toxic AHPND isolates of *V*. *parahaemolyticus*. It is composed by 12 genes and is located in chromosome II. Our results show that the ILI strain does not contain genes with an identity percentage greater than 50%. An additional analysis of genes in the vicinity of T3SS2 resulted in the identification of only two downstream genes as VdILI_04698 and VdILI_04699, which are also located on chromosome II. Furthermore, is important to mention that none of the strains analyzed encode TDH/TRH toxin on T3SS2, except for the clinical isolate RIMD2210633 (Table S7).

**Fig. 8:**
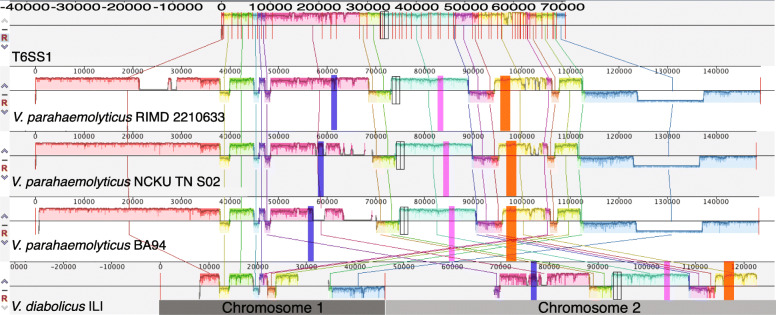
T6SS1 in different species and strains. A multiple alignment of the T6SS1 region and 35Kb upstream and downstream were evaluated for 4 reference genomes. The figure shows the collinear blocks identified between the clinical strain (RIMD 2210633), the one that does not produce AHPND (NCKU_TN_S02) and the one that produces AHPND (BA94) of *V*. *parahaemolyticus*, and the strain of *V*. *diabolicus* ILI. Each gene from the T6SS1 is represented in the top segment with different colors, conservation of each gene and the upstream and downstream regions can be traces by the vertical lines. The ILI strain contains at least partially T6SS1, and the other part is found on chromosome II, which is why we call it T6SS2B. All V. parahaemolyticus strains analyzed in this study contain a highly conserved T6SS1. T6SS1 in ILI contains the effectors described for V. parahaemolyticus by Salomon et al. [47] shown in blue, purple, and orange boxes

Regarding T6SS1 (42 genes), our analysis revealed that the non-AHPND isolates (30/42 genes for *V*. *parahaemolyticus* NCKU TN S02), a toxic AHPND isolate (34/42 genes for *V*. *parahaemolyticus* BA94C2), and the ILI strain contained at least partially the T6SS1 (34/42 genes), but in ILI is located on chromosome II, which is why we named it T6SS2B (Fig. 8; Table S7). This region was also found on chromosome II of the *V*. *diabolicus* strain FDA ARGOS 105. Salomon et al. [47] characterized the T6SS1 antibacterial activity of the RIMD2210633 strain and showed that it is mediated by at least three effectors. We further searched for the three *Vibrio parahaemolyticus* (VP) effectors of T6SS1 and were able to identify them in ILI’s genome (Fig. 8): *V*. *parahaemolyticus* RIMD2210633_01364 (VdILI_03751, Identity 99.21%, Coverage 100%) and *V*. *parahaemolyticus* RIMD2210633_01391 (VdILI_03724, Identity 91.98%, Coverage 19%) within the T6SS1 and effector *V*. *parahaemolyticus* RIMD2210633_01397 (VdILI_03720, Identity 96.43%, Coverage 91%) was present in chromosome I.

The T6SS2 (containing 22 genes, *V*. *parahaemolyticus* RIMD2210633_040- *V*. *parahaemolyticus* RIMD2210633_04059) is also highly conserved in *V*. *parahaemolyticus*; it is located on chromosome II and found in pathogenic and non-pathogenic isolates of *V*. *parahaemolyticus* (Table S7). An equivalent cluster with a high number of genes (20/22) was identified on *V*. *diabolicus* ILI strain on chromosome II (named T6SS2A); the genes were present in ILI with an average percent identity of 73.29%. In comparison, when evaluating 10 genes upstream and downstream from T6SS2A in *V*. *parahaemolyticus* RIMD 2210633, a total of 14/20 genes were present in ILI with an average percent identity of 86.69%.

## Discussion

Shrimp is one of the most important products for aquaculture worldwide. However, due to the massive scale in which shrimp farming is performed, it becomes difficult to control for environmental variables, in particular the presence of pathogens. Currently, AHPND is one of the major risk factors in shrimp farming [2]. Methods such as antibiotics prove to be expensive and unspecific targeting beneficial bacteria as well; thus, probiotics are an important alternative to combat infectious diseases. To date there is very limited information of the effect of AHPND in the shrimp microbiome [48], and existing studies have focused on intestinal microbiota. Even though the contribution of intestinal microbiota on the impact of AHPND disease is not clear, the authors highlight the fact that the diversity on the intestine is higher than on the hepatopancreas, but similar to the one found in sediment. It is known that the immune related organs such as hemocytes, hepatopancreas and gills, are important in the response to AHPND disease [49]. Also, the colonization of the stomach appeared to be the initial step of the infection for *Vibrio* causing AHPND [49]. Additionally, AHPND mainly affects the hepatopancreas and stomachs of infected shrimp; nevertheless, characterizing the microbiota from the shrimp’s stomach has not been a main focus to understand the changes in microbiota responses. Therefore, we examined microbiome alterations in response to pathogenic bacteria producing AHPND, in stomach and hepatopancreas of shrimp.

We identified a significant loss of diversity in the microbiota of hepatopancreas and stomach associated with AHPND (*P* < 0.001, Kruskal-Wallis test). Our results contrast with some minimal effects observed in the intestinal microbiota of shrimp after WSSV infection [50] and AHPND [48]. Thus, we consider that the abundance and composition of the microbiota are influenced as a function of the organ in which it is located. When looking at the effect of the probiotics in the community structure, we observed that the microbiota was closer to the healthy state than the infected configuration of the microbiome, with ILI being the most similar to the microbiota of healthy animals, maintaining a phylum-level diversity including Proteobacteria, Firmicutes, and Tenericutes, which are also the main components of intestinal microbiome in other species, such as crab and black tiger shrimp [50, 51]. In this study, we show that in AHPND infected shrimp, Proteobacteria (primarily order Gammaproteobacteria) was the most dominant phylum and increased in abundance when compared to healthy shrimp and the ILI probiotic treatment.

Interestingly, we identified members from the Tenericutes phylum, in particular those assigned to the genus Mycoplasmatales, as members of the healthy microbiome. Four ASVs from Mycoplasmatales belonged to the genus *Mycoplasma*, being the most abundant taxonomic group of the healthy stomach’s microbiome. This taxon is a common microbiome member in corals like scleractinians and gorgonians [52, 53]. Studies on the role of *Mycoplasma* spp. in corals and in some cnidarians [52, 54] have suggested that it would be harmless commensals or endosymbionts with the host; however, its specific role within the shrimp microbiome is unknown. Specific strains of *Mycoplasma* have been associated with healthy shrimp [55, 56], potentially suggesting that it has a function in host health and recovery from disease states.

We observed an increase in Proteobacteria in infected animals, in particular, most ASVs associated with disease were associated with the Vibrionales order, mainly the Vibrionaceae family. Given that *16S rRNA* gene sequences have a high degree of similarity within the Vibrionaceae, it is impossible to discriminate with precision which ASVs are associated with the pathogen (*V*. *parahaemolyticus*) or with the probiotic (ILI strain) or any other *Vibrio* present in the microbiome. However, the presence of multiple ASVs associated to this family suggest that not only the pathogenic strain responsible of the disease is increasing in abundance, but potentially other taxonomically related Vibrios are increasing as well in consequence of the niche made available by the pathogenic strain. The alignments of ASVs suggested that Vibrionales 1 and 2 can be used as the major biomarkers in the microbiomes of the AHPND-infected shrimp in hepatopancreas, due to the close relationship with species that have been characterized as pathogenic, specifically in the *Harveyi* clade; while *Mollicutes* species could be used as a potential indicator of health or recovery in microbiomes isolated from individual shrimp stomachs.

Surprisingly, some members of the order Vibrionales and Rhodospirillales appeared as associated to healthy individuals. Some of these bacteria have shown antagonistic effects against different bacterial pathogens including known coral pathogens as *Vibrio sp*. [52]. Rhodospirillales species have been reported as probiotics before, but this could demonstrate that these ASVs could be protective against pathogenic *Vibrio* and that it could improve the survival and immunity of shrimp. Furthermore, we were able to observe that the use of bacterial probiotics, such as ILI, can modulate the host microbiota and could contribute as a stimulant for several species that have probiotic potential by inhibiting gastrointestinal colonization of pathogens, as found in this study.

The significant protective effect that the ILI strain showed, and its similarity, at least by *16S rRNA* gene sequence, to potential pathogens, led us to further study the genomic composition and phylogenetic relatedness of this strain. The comparative genomics approach used confirmed inconsistencies in the species classification, where not only the ILI strain was mistakenly classified but other strains had similar errors, likely due to the lack of complete genomic information at the time when the species were described. Current genomic tools and available genomes allowed us to classify them as *V*. *diabolicus*.

The genomic repertoire of ILI’s genome includes cytochrome peroxidase and other cytochromes for reduction of oxygen to hydrogen peroxide, and superoxide for tolerating high oxygen concentrations, and multiple catalase genes. For example, the Bcp-type encodes a functional thiol peroxidase and plays a role in the precise control of H_2_O_2_ levels. The H_2_O_2_ serves as an intracellular messenger at low concentrations, and it induces cell death at higher concentrations. Besides, the host’s immune system will use reactive oxygen species against pathogenic bacteria, which shows the bacteria’s need for an adequate detoxifying system [57]. These results are compatible with the ones reported by Hasan et al. [58], where the authors suggested that the ability to scavenge endogenous hydrogen peroxide was absent in the other *Vibrio* genomes but was present in the genome of *V*. *antiquarius* EX25. Furthermore, Yévenes et al. [59] reported that Vibrio sp. ArtGut-C1 strain has the ability to accumulate polyhydroxybutyrate (PHB), suggesting that *V*. *diabolicus*, *V*. *antiquarius*, and *V*. *alginolyticus* could potentially produce the polymer as they also have PHB genes. It is an interesting finding given that this biodegradable polymer could have anti-pathogenic activity, particularly in the marine larviculture phase. Sequencing of the genome identified genes that confer resistance to antibiotics, in addition to genes that improve the fitness of the organism.

The fact that ILI can acquire the causative plasmid for AHPND shows the high capacity of *Vibrio* species for HGT, which may be used to occupy different niches. The acquisition may vary in the aquatic environment and it may be influenced by the genetic background. ILI strain harbors hemolysins, T3SS, and T6SS that are considered factors associated with virulence in pathogenic *Vibrio* species. We found a certain level of presence and conservation of such secretion systems in ILI; however, the variation compared to the pathogenic strains prevents us from concluding regarding the functionality of such systems. The maintenance of these features in non-pathogenic bacteria could be evidence that secreted toxins are evolutionarily ancient features that may play a larger role in environmental fitness [58].

Pathogens use a specialized set of T3SS translocator proteins to establish virulence in the host cell since it directly delivers bacterial virulence factors, called effectors, into the cytoplasm of host cells in order to exert various functions [60]. Wang et al. [61] determined that four effectors (Vop Q, Vop S, VPA0450 and Vop R (VP1638)) located on T3SS1 of *V*. *parahaemolyticus* are involved in the cytotoxic process, causing autophagy, cell rounding, and finally death. Even though the main cluster of T3SS1 in the ILI strain is structurally similar to the one previously characterized in *V*. *parahaemolyticus*, it does not possess the four effectors described before (Table S7).

Similarly, a homology analysis indicated that the T6SS1 effectors (VdILI_03751, VdILI_03724 and VdILI_03720) are present in different *Vibrio* species, including *V*. *alginolyticus*, *V*. *campbellii*, and *V*. *cholerae* [62]. These effectors were described as necessary for the adhesion of *V*. *parahaemolyticus* to cells and are involved in intracellular trafficking and vesicular transport. The three effectors contain the conserved MIX motif that is found in proteins with predicted cytotoxic domains, including VgrG and PAAR-repeat-containing protein [63]. Li et al. [18] evaluated whether T6SS2 (equivalent toT6SS2A in ILI strain) activates under similar conditions in all types of bacterial strains tested and found that various strains tested under the same conditions did not show T6SS2 activity. They concluded that besides temperature, activation can be linked to other factors. Furthermore, both T6SSs have been defined as required for virulence or survival of a bacterium in a eukaryotic host. However, Weber et al. [64] reported that T6SS in *V*. *anguillarum* regulates stress response, suggesting that T6SS has an ecological function besides other potential pathological function, as can be the case of the ILI strain. Thus, altogether, our data indicates that virulence is multifactorial in the *Harveyi* clade and the sources of virulence could be associated to specific hosts since many of these genes can be involved on the survival of the bacteria in different environments.

The ILI plasmid (340 Kbp) has not been described in any previous report, but it could provide important functions in the probiotic capacities that this strain exhibits. It is intriguing that no other reported pathogenic *Vibrio* strain contains a plasmid with similar gene content. The majority of large plasmids that are associated with *Vibrio* species have been characterized to be highly virulent against different hosts. Interestingly, we did not find any potential virulence factors encoded in the ILI plasmid; thus, the role of the plasmid in the bacteria is not yet clear. Further studies on the role of the plasmid in the ILI strain could clarify its function and potentially shed light on whether it contributes to ILI’s probiotic capabilities.

## Conclusions

The use of probiotics in aquaculture is a practical alternative to promote animal health and prevent disease. We characterized the microbiome alterations generated by a successful probiotic that could control pathogenic populations in shrimp’s gastrointestinal tract and stimulate survival in aquaculture. *V*. *diabolicus* ILI strain is a bacterial strain isolated from the environment in a larvae shrimp culture that has shown antimicrobial activity against many pathogenic strains of different *Vibrio* species [13]. Supplementation of ILI to *P*. *vannamei* not only decreased the abundance of Proteobacteria ASVs, but also maintained a healthy-like microbial community in the shrimp gastrointestinal tract after being challenged with AHPND causing bacteria. Our findings suggest that ILI strain can likely be used as a probiotic to reduce the population of pathogens for AHPND in the shrimp and to enhance survival and resistance against this emerging disease in shrimp aquaculture, without the risk of becoming a pathogenic organism.

## Supplementary Information

**Additional file 1: Figure S1.** Shrimp cumulative mortalities after feeding the animals with the probiotics for a month’s period and being challenged with *Vibrio parahaemolyticus* BA94C2 strain. Negative control was TSB 2% NaCl. Mortalities were followed for up to 3 days. It is observed a delay on the onset of mortalities and a reduction in cumulative mortality as an effect of the probiotic treatment.

**Additional file 2: Figure S2.** Rarefaction curves for samples obtained from all treatments. Graphs represent the Observed OTU metric subsampled at different sequencing depths (500 – 4000) with 10 different replicates. Saturation is shown at approximately 1500 sequences for all samples.

**Additional file 3: Figure S3.** Graphical visualization of the alignment of V4 region of the *16S rRNA* gene from 38 related Vibrio species. Variations from the reference sequence are highlighted along the length of the alignment for species of the different known Vibrio species. Although taxonomical assignments would assign four ASVs to the order Vibrionales, it is possible to observe clade specific SNPs in particular location of the sequences. The set of ASV recovered from the microbiota experiment are highlighted in red.

**Additional file 4: Figure S4.** Evaluation of possible biomarkers. The abundances of ASVs in the stomach of infected, healthy, and probiotic-treated shrimp were compared. (A) The ASVs identified as discriminatory for AHPND-infected shrimp in the stomach were Vibrionales 1, 3, 2 and 4. (B) The ASVs identified as discriminatory for healthy shrimp in the stomach were Mollicutes 2 and 3, Acetobacter and *Photobacterium damselae*. Interestingly, we found that these ASVs showed a significant increase in ILI treatment compared to healthy animals. Samples from C0 and C1 correspond to 5 samples, while for C2 and C3 is a single pooled sample.

**Additional file 5: Table S1.** Primers sequences used in the study. AP4 (1 and 2) are for the detection of Pir-*VP* genes. Vpir A is for the detection of Pir-A gene while Vpir B is for the amplification of Pir-B gene. Length represents the length of the amplicon generated by each pair of primers. **Table S2.** ILI’s genome protein annotation using Prokka against the NCBI’s database. **Table S3.** ILI’s large plasmid proteins predicted by Prokka against the NCBI’s database with their corresponding functions and ID. The number of ASVs detected per sample is plotted on the axis of Observed OTUs. Function is assigned when hits with an e-value < 1e- 06 were obtained. **Table S4.** Genomes used to for genomic similarity analysis. The genomic identity between ILI and the selected genome is shown in the last column of the table. **Table S5.** GenBank accession numbers and genomic characteristics of genomes used for the phylogenetic reconstruction of the ILI strain. **Table S6.** DDH, ANIb and TETRA values. In silico DNA-DNA hybridization (DDH) was calculated only between the ILI strain and the other evaluated strains. ANIb and TETRA were calculated for all possible comparisons. A heatmap representation of the values can be observed on Figure 3. **Table S7.** Virulence factors identified in the secretion systems of the strain *V. diabolicus* ILI. They were compared with the genomes of the V. parahaemolyticus RIMD 2210633 strain, the *V. parahaemolyticus* NCKU TN S02 strain, the *V. parahaemolyticus* BA94C2 strain and the *V. diabolicus* FDA 105 strain. The table includes the respective name of the genes for each genome, the gene product, the percent identity, and the percent coverage. Comparisons based on percentage of identity and coverage included are shown in key color.

## Data Availability

All relevant data are within the paper and its additional files. Raw sequence data from this experiment were uploaded to the Sequence Read Archive (SRA) under Bioproject accession number PRJNA580262.
