# Supplementary figures and images for: Microbial community characterization of shrimp survivors to AHPND challenge test treated with an effective shrimp probiotic (Vibrio diabolicus)
Source: Microbiome. 2021 Apr 12;9:88. doi: 10.1186/s40168-021-01043-8 (PMC8042889; doi:10.1186/s40168-021-01043-8)

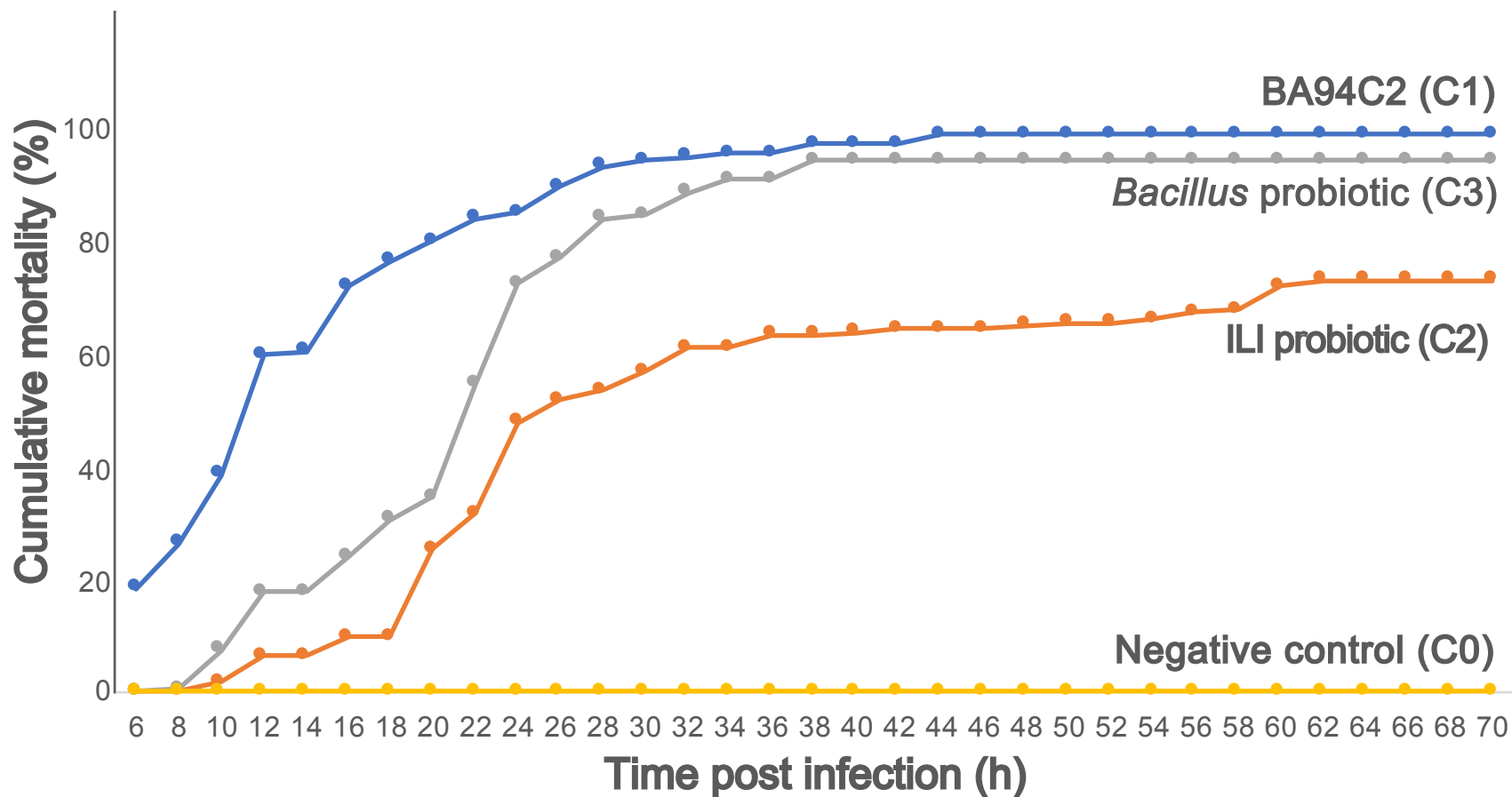

Supplement: Supplementary file 2 — Additional file 1: Figure S1. Shrimp cumulative mortalities after feeding the animals with the probiotics for a month’s period and being challenged with Vibrio parahaemolyticus BA94C2 strain. Negative control was TSB 2% NaCl. Mortalities were followed for up to 3 days. It is observed a delay on the onset of mortalities and a reduction in cumulative mortality as an effect of the probiotic treatment. [file 40168_2021_1043_MOESM1_ESM.pdf]

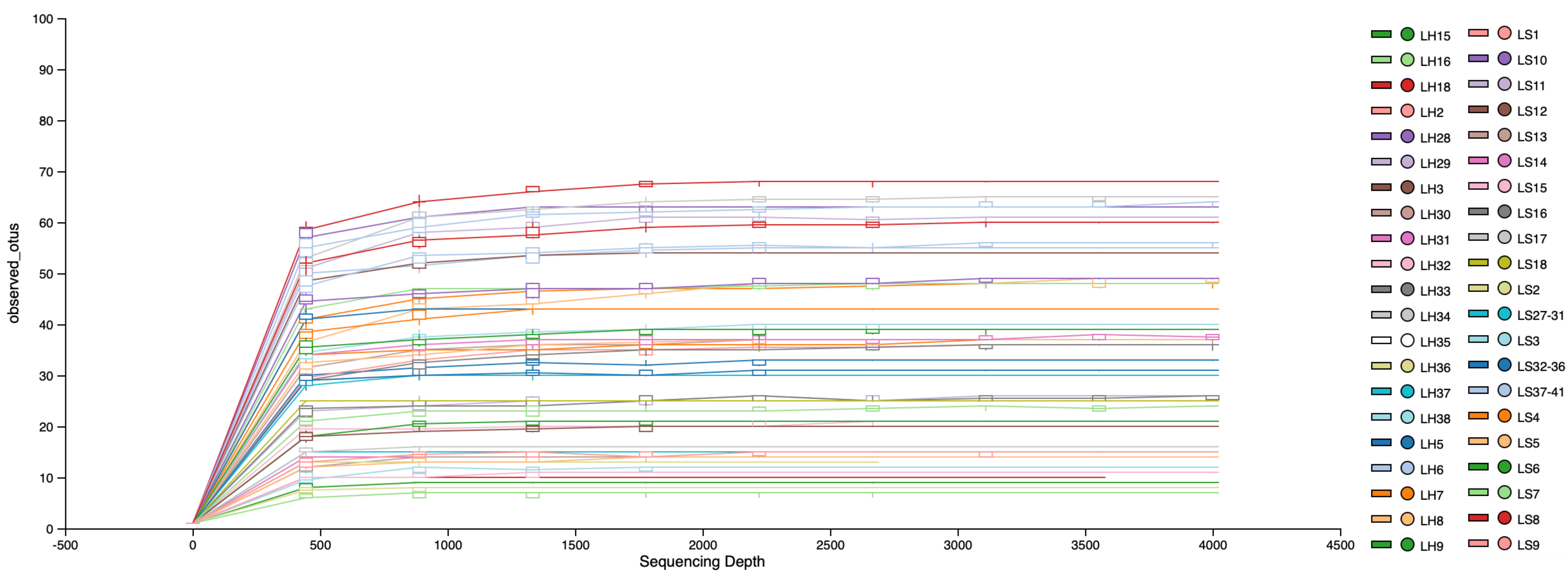

Supplement: Supplementary file 3 — Additional file 2: Figure S2. Rarefaction curves for samples obtained from all treatments. Graphs represent the Observed OTU metric subsampled at different sequencing depths (500 – 4000) with 10 different replicates. Saturation is shown at approximately 1500 sequences for all samples. [file 40168_2021_1043_MOESM2_ESM.pdf]

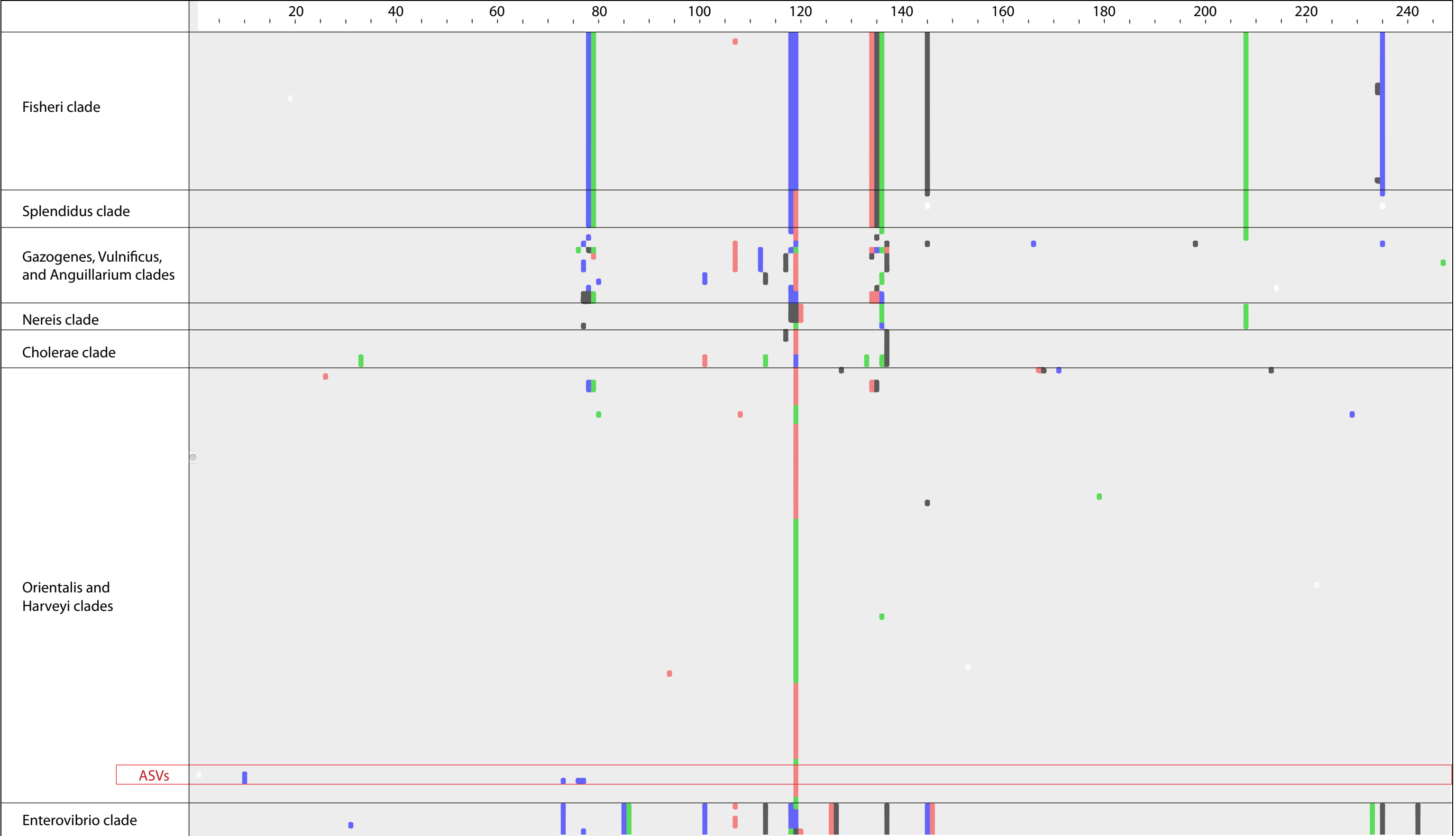

Supplement: Supplementary file 4 — Additional file 3: Figure S3. Graphical visualization of the alignment of V4 region of the 16S rRNA gene from 38 related Vibrio species. Variations from the reference sequence are highlighted along the length of the alignment for species of the different known Vibrio species. Although taxonomical assignments would assign four ASVs to the order Vibrionales, it is possible to observe clade specific SNPs in particular location of the sequences. The set of ASV recovered from the microbiota experiment are highlighted in red. [file 40168_2021_1043_MOESM3_ESM.pdf]

A

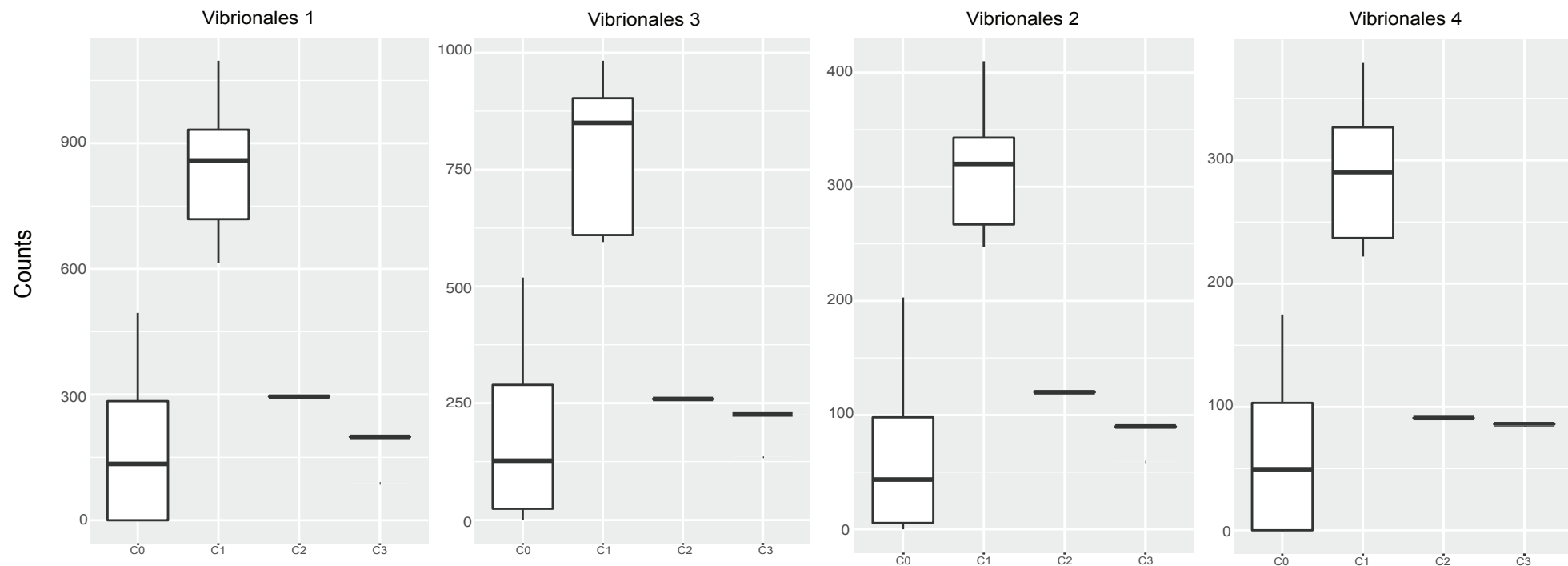

B

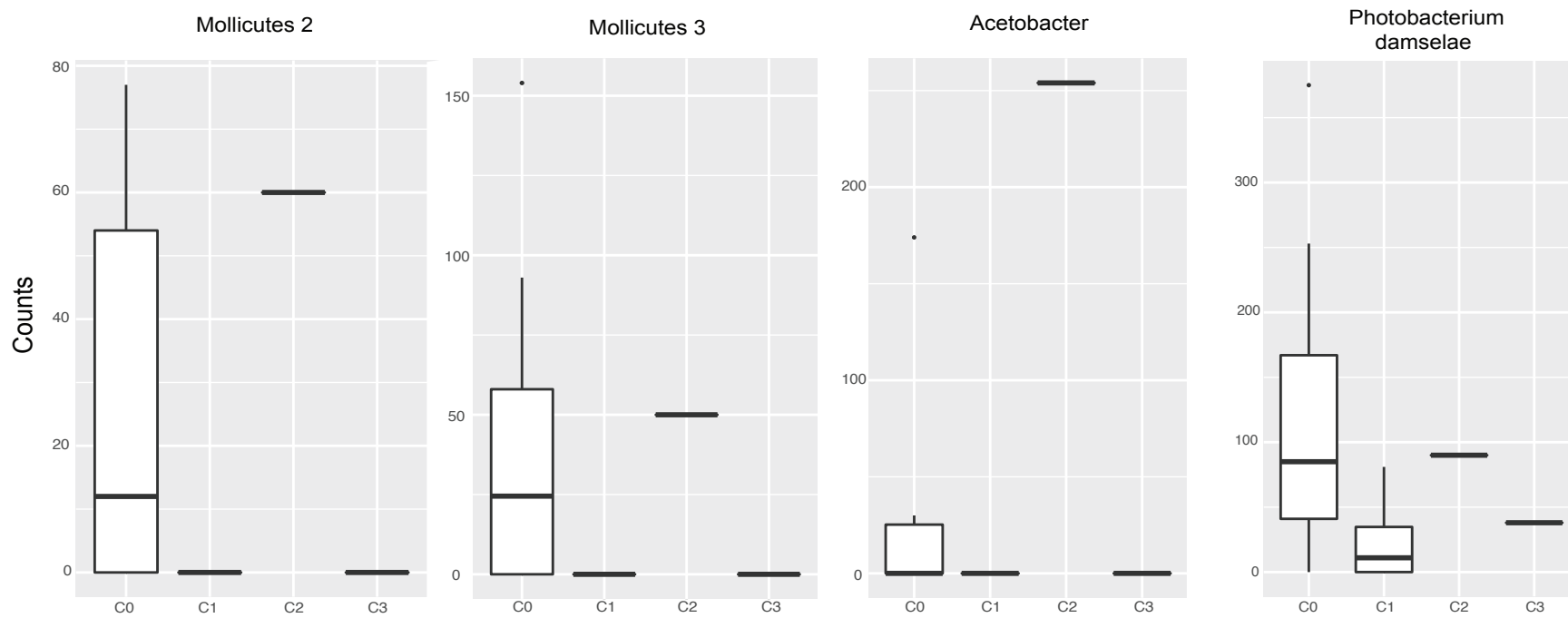

Supplement: Supplementary file 5 — Additional file 4: Figure S4. Evaluation of possible biomarkers. The abundances of ASVs in the stomach of infected, healthy, and probiotic-treated shrimp were compared. (A) The ASVs identified as discriminatory for AHPND-infected shrimp in the stomach were Vibrionales 1, 3, 2 and 4. (B) The ASVs identified as discriminatory for healthy shrimp in the stomach were Mollicutes 2 and 3, Acetobacter and Photobacterium damselae. Interestingly, we found that these ASVs showed a significant increase in ILI treatment compared to healthy animals. Samples from C0 and C1 correspond to 5 samples, while for C2 and C3 is a single pooled sample. [file 40168_2021_1043_MOESM4_ESM.pdf]
